# Unraveling the Significance of DGCR8 and miRNAs in Thyroid Carcinoma

**DOI:** 10.3390/cells13070561

**Published:** 2024-03-22

**Authors:** Lia Rodrigues, Arnaud Da Cruz Paula, Paula Soares, João Vinagre

**Affiliations:** 1 Instituto de Investigação e Inovação em Saúde da Universidade do Porto (i3S), Rua Alfredo Allen, 4200-135 Porto, Portugal; lrodrigues@i3s.up.pt (L.R.); arnaud.paula@i3s.up.pt (A.D.C.P.); psoares@ipatimup.pt (P.S.); 2 Instituto de Patologia e Imunologia Molecular da Universidade do Porto (Ipatimup), Rua Júlio Amaral de Carvalho, 4200-135 Porto, Portugal; 3 Departamento de Patologia, Faculdade de Medicina da Universidade do Porto, Alameda Prof. Hernâni Monteiro, 4200-319 Porto, Portugal; 4 Escola Superior de Saúde do Instituto Politécnico do Porto, Rua Dr. António Bernardino de Almeida, 4200-072 Porto, Portugal

**Keywords:** microRNAs, thyroid cancer, DGCR8, E518K

## Abstract

MicroRNAs (miRNAs) act as negative regulators for protein-coding gene expression impacting cell proliferation, differentiation, and survival. These miRNAs are frequently dysregulated in cancer and constitute classes of blood-based biomarkers useful for cancer detection and prognosis definition. In thyroid cancer (TC), the miRNA biogenesis pathway plays a pivotal role in thyroid gland formation, ensuring proper follicle development and hormone production. Several alterations in the miRNA biogenesis genes are reported as a causality for miRNA dysregulation. Mutations in microprocessor component genes are linked to an increased risk of developing TC; in particular, a recurrent mutation affecting *DGCR8*, the E518K. In this review, we explore these novel findings and resume the current state-of-the-art in miRNAs in thyroid carcinomas.

## 1. Introduction

### 1.1. miRNA Biogenesis Pathway

**Figure 1:**
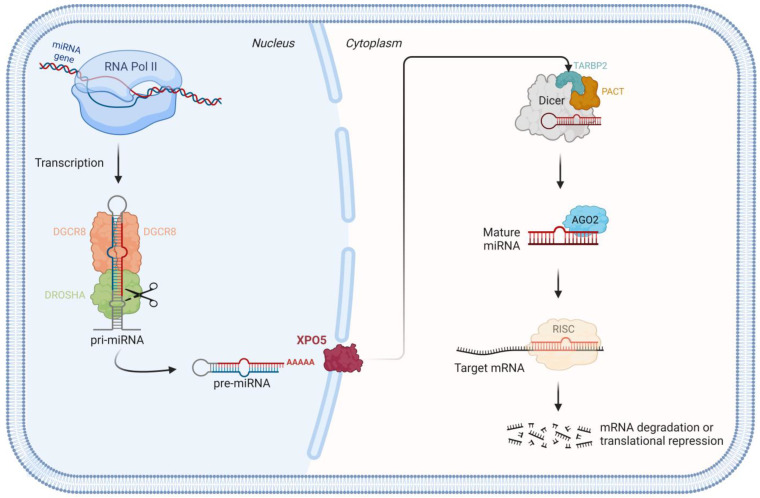
miRNA biogenesis pathway: miRNA genes are transcribed into the nucleus by RNA pol II into primary structures—pri-miRNA. The microprocessor complex composed of DROSHA and its binding partner DGCR8 cleaves the pri-miRNA into a hairpin pre-miRNA structure. The pre-miRNAs are then exported to cytoplasm by XPO5 where DICER processes the pre-miRNA into mature miRNA. The mature form is incorporated into RISC which will guide the mature miRNA to target mRNAs, regulating gene expression post-transcriptionally by mRNA degradation or translational repression. The figure was created with BioRender.com.

MicroRNAs (miRNAs) are a class of small, non-coding, single-stranded RNAs that are responsible for the regulation of gene expression at a post-transcriptional level and function as negative regulators of gene expression [1,2]. The miRNA genes are encoded throughout the genome of most eukaryotic organisms and actively transcribed by RNA polymerase II (RNA pol II) into long, poly-adenylated, and capped primary miRNAs (pri-miRNAs) in the nucleus [2,3,4]. These structured RNAs are then subjected to processing by a 364 kDa trimeric nuclear complex—microprocessor protein complex—constituted of two DiGeorge Critical Region 8 (DGCR8) proteins (86 kDa) attached to a Drosha Ribonuclease III (DROSHA) (159 kDa) protein. Following processing, the pri-miRNAs are converted into approximately 60 to 70 nucleotide (nt) hairpin-shaped intermediates, the so-called precursor miRNAs (pre-miRNAs), as represented in Figure 1 [3,4,5]. Biochemical evaluation of the microprocessor complex in human cells determined that DROSHA can form two different complexes: a smaller complex, comprising DROSHA and its partner DGCR8 that constitutes the minimal catalytically active complex, and a larger complex that contains additional accessory RNA-binding proteins, such as RNA helicases, heterogeneous nuclear ribonucleoproteins, and other associated proteins that can regulate its activity [6].

The pre-miRNAs are consequently exported to the cytoplasm by the nuclear transport receptor exportin 5 (XPO5), where the Dicer ribonuclease III (DICER) cleaves to the base of the loop to generate about 21 to 24 nt double-strand miRNA duplexes [3]. In humans, DICER interacts with two closely related proteins—TARBP2 subunit of RISC loading complex (TARBP2) and protein kinase RNA activator (PACT), which are not required for activity, but have been implicated in defining the cleavage site and facilitating the formation of RNA-induced silencing complex (RISC) [6]. The abovementioned proteins DROSHA, DGCR8, DICER, TARBP2, and PACT are double-stranded RNA-binding proteins (DRBP) consisting of 1 to 3 RNA-binding domains that allow the binding of miRNAs or the interaction with another DRBP [7].

The duplex is unwound with one strand being preferentially selected due to its stability of the base pairs at the 5′ end of the duplexes, while the remaining one is degraded [4]. The selected strand binds to the Argonaute (AGO2) proteins to generate the final and mature form of miRNA [4,6]. The mature miRNA is a unique double-stranded RNA consisting of complementary RNA chains of 21–23 nts with 2-base overhang [8]. This mature miRNA is incorporated into a ribonucleoprotein complex, RISC [4]. The miRNAs regulate gene expression post-transcriptionally, where they act as negative regulators of coding gene expression, by guiding the RISC to their cognate sites at the 3′-untranslated region (UTR) of target mRNAs [9,10]. Although most miRNAs target the 3′ UTR of the mRNA targets, some miRNAs are capable of interacting with the coding sequences of the target genes [11]. The target mRNA will be initially subjected either to cleavage or translation repression by inhibiting ribosomal access, depending on whether the miRNA–mRNA pairing is perfect or not, respectively [3,9,12,13]. It is postulated that more than 50% of human mRNAs may be influenced by miRNA-mediated regulation, suggesting that miRNAs may be involved in all biological processes [12,14].

The canonical miRNA biogenesis pathways driven by the RNase III enzymes DROSHA and DICER have been challenged by the discovery of an unexpected variety of alternative mechanisms, such as the spliceosome, that generates functional miRNAs [6]. This DROSHA and DGCR8-independent non-canonical pathway is represented by the processing of miRNAs located inside intronic regions (miRtrons) where the introns excised by the spliceosome are directly cleaved and loaded by DICER to generate mature miRNAs [6].

Numerous regulatory pathways of miRNAs are described, with the most prominent one being the LIN28-DIS3L2-*let*-7 pathway, in which LIN28 protein negatively regulates *let*-7 miRNA synthesis by reducing the cleavage activity of DROSHA and DICER and facilitating the poly-uridylation resulting in degradation by DIS3L2 [15,16]. Regulatory post-translational modifications (PTMs) such as phosphorylation, ubiquitylation, and SUMOylation of miRNA biogenesis factors can influence miRNA processing. These modifications establish a connection between miRNA expression and cellular signaling pathways [15,16,17].

Non-canonical functions of miRNA biogenesis proteins are also described, with functions independent of the miRNA biogenesis pathway, and related to various biological processes such as development, transcriptional regulation, RNA processing, and maintenance of genome integrity [18].

### 1.2. miRNAs as Powerful Biomarkers in Cancer

The miRNAs were identified as key molecules that regulate cellular processes such as cell proliferation, apoptosis, growth, senescence, adhesion, invasion, and migration by targeting the mRNAs involved in a variety of cancer-related signaling pathways [14,19]. Hence, alterations in the expression levels of mRNAs through miRNA dysregulation are postulated to affect the initiation, development, and progression of cancer [20]. Previous studies demonstrated that DNA methylation and histone modification have a major control function in miRNA transcription [21]. The fact that the majority of miRNA genes are located at chromosomal fragile sites, common breakpoint sites, or within regions of deletion or amplification that are generally altered in tumors, highlights their relevant role in human cancer [4,22]. The miRNA genes are deemed to function as both oncogenes and tumor-suppressor genes, with their expressions being associated with several types of cancer [2]. The miRNA expression is dysregulated in human cancer not only by amplification or deletion of miRNA genes, but also through various mechanisms such as abnormal transcriptional control of miRNA expression by oncogenic transcription factors, epigenetic changes such as chromatin remodeling through methylation of promotor sequence, and/or defects in the miRNA processing machinery [23,24]. Mutations in miRNA target sites that lead to incorrect mRNA recognition may induce severe phenotypic consequences and may promote carcinogenesis [24].

More than 2000 miRNAs have been identified in humans [24]. As presented by Voorhoeve et al. [20], miRNAs can be inferred as “the good, the bad and the ugly”: “the good” are miRNAs as innocent bystanders in the oncogenic transformation process, whose expression profile can be a surrogate for cancer diagnosis and prognosis; “the bad” are miRNAs that are causally linked to tumorigenesis and modify tumor-suppressor or oncogenic pathways; and “the ugly” are miRNAs representing the ones whose inappropriate loss or gain destabilizes the cellular identity of a tumor, resulting in enhanced phenotypic variability and tumor progression [20].

**Figure 2:**
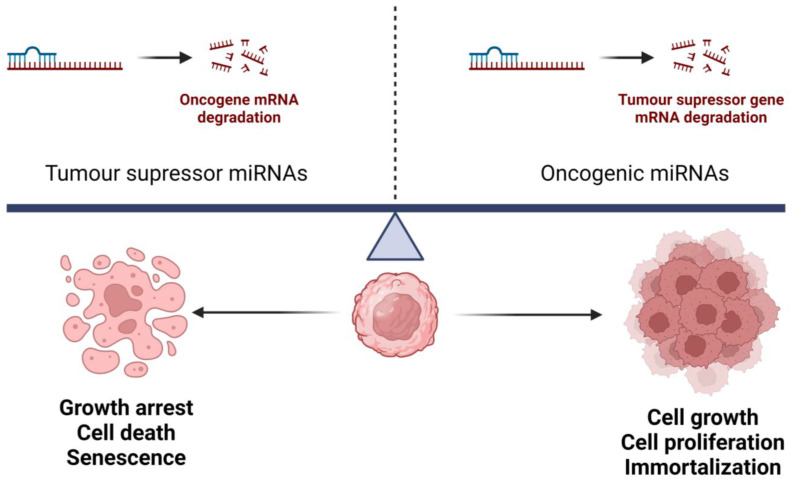
Schematic representation of the balance between oncogenic miRNAs and tumor-suppressor miRNAs. Downregulation of tumor-suppressor miRNAs could result in overexpression of oncogene mRNAs by reducing degradation. In turn, overexpression of oncogenic miRNAs could result in the downregulation of tumor-suppressor gene mRNAs by increasing mRNA degradation. Both processes have an impact on hallmarks of cancer such as cell proliferation, immortalization, and angioinvasion. The figure was created with BioRender.com.

The analysis of global miRNA expression in cancer patients pointed to different patterns of miRNA overexpression or downregulation in cancer when compared to their normal counterparts, impacting cell proliferation and apoptosis [4,25]. This miRNA dysregulation exerts a pro-oncogenic effect, as the overexpression of one miRNA may act to inhibit the protein translation of a tumor-suppressor gene, while the downregulation of another miRNA may increase the protein level of an oncogene [14]. Overexpression of miRNAs—through amplification or loss of epigenetic silencing of a gene encoding an oncogenic miRNA—could result in the downregulation of tumor-suppressor genes by targeting one or more different mRNAs (oncogenic miRNAs); and downregulation of miRNAs—through deletion, subtle mutation, or epigenetic silencing—could result in the upregulation of oncogenes (suppressor miRNAs) with subsequent effects on cell proliferation, apoptosis, angioinvasion, and other carcinogenic actions, as represented in Figure 2 [20,25,26,27]. It has been previously proposed that the balance between oncogenic versus tumor-suppressive miRNAs acting within transcription factor—miRNA regulatory networks, influences the multistep process of neoplastic transformation and their stress response [20]. The role of these oncogenic or suppressor miRNAs is exerted through the modulation of cancer-related signaling pathways such as the mitogen-activated protein kinase (MAPK) and PI3K/Akt/mTOR [14,25].

The major evidence linking miRNAs to cancer is their large variation in expression profiles when comparing malignant and benign cells [23]. An overall downregulation of miRNA expression is detected in tumoral tissues, suggesting a higher share of tumor- suppressor miRNAs, which may indicate a reduced cell differentiation state, an additional hallmark of human cancer [14,20]. Moreover, poorly differentiated tumor samples present a lower miRNA expression when compared to more differentiated ones, consistent with the hypothesis that a higher global miRNA expression is associated with cellular differentiation [4]. Similarly to protein-coding genes, miRNAs are submitted to epigenetic modulation such as disruption of DNA methylation and histone modification patterns [28].

MiRNAs are described to be dysregulated not only in tumors but also in stromal cells, emerging as crucial regulators of tumorigenesis [28]. Tumoral cells use their own miRNA repertoire, by several mechanisms of miRNA transference, to hijack tumor-promoting functions of immune cells [28]. A variety of miRNAs are implicated in the tumor immune surveillance as well as tumor immune escape, being reported to interact with many immune checkpoint proteins [17]. Therefore, miRNAs loaded in extracellular vesicles can affect not only the progression and metastasis but also resistance to therapy and tumor microenvironment [17].

The miRNA expression profiling of human tumors has identified signatures associated with diagnosis, staging, prognosis, and response to therapy [29]. Hence, manipulating miRNA expression levels may serve as a potential therapeutic strategy against cancer [20,30]. This miRNA modulation, consisting of the restoration of downregulated suppressor miRNAs with synthetic oligonucleotides and the inhibition of overexpressed oncogenic miRNAs by miRNA antagonists, can be conducted by several approaches [20,21,30,31].

As miRNA machinery component defect is one of the mechanisms which miRNA dysregulation relies on, the effect of global miRNA downregulation was previously reproduced in vitro by knocking down miRNA machinery [14]. The regulation of miRNA machinery components can directly influence the expression patterns of various genes by regulating mRNA expression—if any miRNA machinery component is dysregulated, miRNAs may be incompletely matured [32]. Studies reported that impaired miRNA processing caused by the aberrant expression of miRNA biosynthesis genes like *DROSHA* or *DGCR8* can noticeably promote tumorigenesis [2]. Not only are alterations in expression described, but also mutations in genes involved in the processing of miRNAs that include *DROSHA*, *DGCR8*, *XPO5*, *DICER1*, and *AGO*. Mutations are also reported both at the somatic and germline level [10,14,33,34]. At germline level, these genes are known to cause at least three distinct genetic syndromes, with clinical manifestations that range from tumor predisposition (*DGCR8* and *DICER1* germline mutations) to neurodevelopmental disorders (*AGO1/2* germline mutations) [35]. Hence, single nucleotide polymorphism (SNP) in *DROSHA* and *DGCR8* genes can affect their structure or expression, resulting in incomplete miRNA processing influencing the expression of the target gene, thereby acting as a risk factor for cancer that will be further discussed [2].

### 1.3. miRNAs in Thyroid Tumorigenesis

The miRNA biogenesis pathway is postulated to hold a key role in the early and proper development of the thyroid gland, as miRNAs are necessary for the accurate establishment of thyroid follicles and hormone synthesis [10,36]. In line with numerous types of human carcinomas, the miRNA expression in thyroid carcinoma (TC) is also found to be perturbed [26]. The miRNA function has been identified as an important driver for tumor development and progression to TC [24]. Most TCs are differentiated follicular cell-derived carcinomas: papillary thyroid carcinoma(s) (PTC(s)), follicular thyroid carcinoma(s) (FTC(s)), and oncocytic carcinoma(s) (OCA(s)). PTC, FTC, and OCA may progress to differentiated high-grade thyroid carcinoma(s) (DHGTC(s)) or poorly differentiated thyroid carcinoma(s) (PDTC(s)); or even to anaplastic thyroid carcinoma(s) (ATC(s)) [37]. Less than 5% of the cells within the thyroid gland are C-cells that give rise to a neuroendocrine tumor—medullary thyroid carcinoma (MTC) [33,38,39]. Some FTCs are supposed to be the progression of a benign thyroid tumor—follicular thyroid adenoma (FTA) [26].

The activation of oncogenes is a known cause of miRNA global dysregulation in thyroid cells [14,36]. An important proliferative pathway in thyroid cells is the MAPK signaling pathway that when activated by oncogenes, i.e., *RET/PTC*, *RAS*, and *BRAF*, promotes sustained cell proliferation, with these mutations being reported in more than 70% of PTCs [14,36]. Evidence suggests that dysregulated activation of the MAPK cascade can increase the genomic instability of TC cells, thus promoting the acquisition of additional somatic mutations during TC progression [24]. Thyroid malignant transformation by MAPK oncogenes is accompanied by global miRNA changes with a marked reduction of suppressive miRNAs and activation of oncogenic miRNAs [14].

The normal thyroid gland expresses higher levels of specific miRNAs that are all downregulated in TC indicating an important tumor-suppressive action of those miRNAs [14,26]. Previous studies suggested a vital role for specific miRNAs as key factors in the development and progression of TC [29]. It has been hypothesized that among all the dysregulated miRNAs, only those that are abundantly overexpressed or strongly downregulated, are involved in thyroid tumorigenesis [4]. The *let*-7 miRNA family, a classical tumor-suppressor miRNA family, highly expressed in the normal thyroid gland regulates the proto-oncogene *RAS*, a component of the MAPK pathway that is frequently mutated in follicular-patterned tumors [14,24]. *Let*-7 was shown to have complementary binding sites in the 3′-UTR of all 3 *RAS* genes (*HRAS*, *KRAS*, and *NRAS*) and it functions to tamper RAS protein levels [27]. Because RAS activation is related to several cancers, *let*-7 downregulation or deletion could be crucial in tumorigenesis [27].

The involvement of miRNAs in the thyroid has recently changed the paradigm for biomarker discovery in TC, suggesting that these small non-coding RNAs could be used to develop, refine, or strengthen strategies for diagnosis and therapeutics of TC [24]. Recent studies described the use of some miRNAs as biomarkers in diagnosis, prognosis, and therapeutic targets of TC, as different subtypes of TC are associated with specific miRNA profiles [26]. However, the miRNAs as biomarkers neglected the heterogeneity between patients, complicating the composition of a transversal signature [40]. The miRNA expression profile is different, not only between normal and tumor tissues but also between different tumor types, stages, primary, and metastatic tumors [22]. Previous studies described that many miRNAs are tissue-specific, in the particular case of the thyroid, a markedly different miRNA profile has been shown between MTCs and follicular cell-derived carcinomas [36]. Expression profiling of miRNAs was found to be likewise able to distinguish tumors with *BRAF*, *RET/PTC*, and *RAS* mutation [24,26,29].

**Figure 3:**
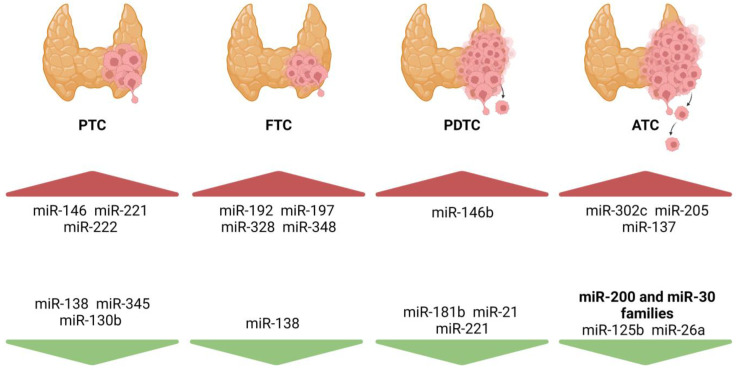
miRNA overexpression (red) and downregulation (green) are able to distinguish tumoral and non-tumoral counterpart tissues and different histotype stratification in TC. Regarding PTC, miR-146 is commonly found upregulated. In FTC, an upregulation of miR-192, -197, -328, and -348 is described when compared to the normal counterpart. PDTC and ATC, which are more likely to metastasize (depicted by the arrow), also present specific miRNAs up- and downregulated, that being the downregulation of miR-200 and miR-30 families strongly associated with ATC. PTC: papillary thyroid carcinoma; FTC: follicular thyroid carcinoma; PDTC: poorly differentiated thyroid carcinoma; ATC: anaplastic thyroid carcinoma. The figure was created with BioRender.com.

It has been described that the differential expression of four miRNAs, namely, miR-100, -125b, -138, and -768-3p, can distinguish benign from malignant thyroid tissue samples [41]. Well-differentiated TC (PTC and FTC) can also be distinguished from ATC by the upregulation of miR-200 and -30 family members, and by downregulation of miR-138 in the differentiated tumors [24]. In PTC, the miR-146, -221, and -222 are consistently upregulated when compared to benign tissue, and miR-138, -345, and -130b were reported to be downregulated in several studies, as depicted in Figure 3 [25,40,42,43]. In FTC, miR-192, -197, -328, and -348 were identified as being upregulated when compared to benign lesions [4,26,40]. In PDTC, miR-146b is highly expressed whereas a downregulation of miR-181b, -21, and -221 was reported [24]. In ATC, miR-302c, -205, and -137 were upregulated with expression levels being more than 60 times higher than in normal tissue, promoting dedifferentiation and aggressiveness, whereas miR-30-d, -30a-5p, -125b, and -26a were found to be downregulated [25,26,40]. The downregulated expression of miRNAs belonging to the miR-200 and miR-30 families is exclusively associated with ATC and is therefore suspected to play key roles in the acquisition of particularly aggressive tumor phenotypes [42]. Interestingly, miRNAs that are commonly upregulated in PTC are not deregulated in ATC, suggesting that their expression decreases in the dedifferentiation process [40]. In MTC patients, increased expression of miR-21, -183, and -375 has been associated with persistent and metastatic disease [42]. Overexpression of miR-125b and -26a in vitro was able to reduce cell growth and proliferation suggesting a possible role in progression and dedifferentiation [26,43]. Besides the dysregulation in expression, mutations in miRNA genes can also lead to cancer predisposition. One example is a SNP affecting miR-146 that was found to contribute to TC initiation [20].

Due to its discriminative capacity, two miRNA-based molecular tests are already available commercially to aid in the diagnosis of indeterminate thyroid nodules on fine-needle aspiration cytology [44]. The miRNA-base tests classify cytologically indeterminate thyroid nodules into benign or suspicious malignancies by miRNA profiling [19]. It has been shown that the concentration of specific circulating miRNAs in the blood is tightly linked to molecular events occurring in those body regions affected by the disease, providing an indirect way of measuring molecular events of diagnostic importance [24]. The miRNAs as biomarkers in minimally invasive molecular tests were demonstrated to be correlated with features like tumor size, multifocality, lymph node metastasis, and staging [24]. Beyond diagnostic markers, miRNAs are potential therapeutic targets, as previous studies have shown the capacity for restoring thyroid function and radioiodine trapping in radiotherapy refractory TC cells [14]. However, it is important to note that miRNAs are also physiologically expressed by cells in different organs, not all of which are involved in a pathological process [24]. Supplements or inhibitors of miRNAs can directly modulate the behavior of cancer cells and control the progression of cancer. MiRNA-based therapies already enter clinical trials to treat tumors, as it could be applied as complementary therapy [45]. Within the context of TC, modulation of the *let*-7 miRNA family presents itself as a prospective alternative therapeutic target, given its high expression in normal thyroid tissue placed side by side with its downregulation in TC. The ability of miRNAs to target multiple key oncogenes influencing cellular pathways make miRNA-based therapies more effective than single gene therapies [21,24,45].

### 1.4. DGCR8, a miRNA Biogenesis Component, Is Dysregulated in Thyroid Tumors

DGCR8 is one of the proteins involved in miRNA biogenesis, being a component of the microprocessor complex. DGCR8 is described as being a specific of canonical miRNA processing rather than another class of small RNAs, contrary to DICER that also generates non-canonical miRNAs and other small RNAs [46,47]. Besides miRNA biogenesis, DGCR8 plays important roles in development, oncogenesis, the exit of mouse embryonic stem cells (ESCs) from pluripotency, the maintenance of heterochromatin organization, and the regulation of nucleotide excision repair and double-strand break repair [48]. As the others DRBP involved in the miRNA biogenesis pathway, DGCR8 is also essential for immune modulation [7,49,50]. PTMs of DGCR8 modulate its function in miRNA biogenesis. Previous studies described that phosphorylation of DGCR8 N-terminal by MAPK/ERK pathway increases its protein stability whereas deacetylation of DGCR8 domains by HDAC1 enhances its affinity with pri-miRNAs. Zhu et al. reported another PTM of DGCR8, where DGCR8 was modified by a small ubiquitin-like modification—SUMOylation. The SUMOylation alters DGCR8 affinity with pri-miRNAs and, consequently, controls the direct function of pri-miRNAs in recognition and repression of target mRNAs, linking the DGCR8 function with tumorigenesis [48,51].

The high expression of DGCR8 was shown to promote the occurrence, development, and metastasis of cancer, including in TC [52]. It has been reported that TGF-β, a cytokine with an important role in promoting proliferation and metastasis in some types of cancers, is positively regulated by DGCR8 [52]. The overexpression of DGCR8 upregulates TGF-β, promoting cell proliferation and metastasis [52]. Moreover, it is described that the knockdown of *DROSHA* leads to the upregulation of *DGCR8* expression at the mRNA level and protein levels, suggesting that not only alterations in *DGCR8* but also in other miRNA biogenesis genes could alter the DGCR8 protein expression [53]. A post-transcriptional DGCR8/DROSHA autoregulatory feedback loop is described, postulating that when DROSHA and DGCR8 levels are elevated in the cell, the microprocessor would cleave and destabilize the *DGCR8* mRNA, resulting in a reduction of DGCR8 levels [53,54]. It has been also reported that the downregulation of *DGCR8* is induced by the microprocessor activity [7]. Other proteins can also control *DGCR8* expression, like the case of the ING1 protein that is functionally linked to the p53 pathway and chromatin regulation. ING1 is responsible to bind to the *DGCR8* promoter and control its transcription by inhibiting the histone acetylation through active recruitment of deacetylation complexes [55]. Mechanisms such as DNA methylation could also be at play in *DGCR8* dysregulation, as previously demonstrated in injured Schwann cells [56]. On the flip side, a reciprocal relationship exists between the N6-Methyladenosine (m6A) of mRNA and DGCR8. In this context, the m6A modification accelerates miRNA processing by facilitating the interaction between DGCR8 and pri-miRNA. This interaction is mediated through the collaboration of m6A methyltransferases, such as METTL3, and m6A recognition factors, such as HNRNPA2B1, with DGCR8 [57]. The presence of a virus is also reported to have an impact on DGCR8 expression, both at RNA and protein levels [7].

Wang et al. [58] demonstrated that the knockout of *DGCR8* in ESCs is unable to impede self-renewal, which is believed to be silenced as differentiation of ESCs occurs. However, the introduction of *let*-7 miRNAs—the family of miRNAs, highly expressed in somatic cells including thyroid cells, can prevent self-renewal. These results suggest that *DGCR8* disruption negatively affects the levels of *let*-7 miRNAs, one of the highly expressed miRNAs in the normal thyroid gland. As the *let*-7 miRNA family targets RAS, a component of the MAPK pathway, the RAS activation through *DGCR8* disruption and consequently *let*-7 downregulation or deletion could be crucial in tumorigenesis [27]. It has been previously reported by Puppin et al. [59] that the expression of miRNA machinery components, such as *DGCR8*, is increased by the presence of *RET* mutations, suggesting that those components are subjected to RET regulation in a RAS-independent manner. Recurrent somatic mutations in the RAS family are reported in FTC, highlighting the role of *DGCR8* in follicular thyroid lesions, a subject that will be further addressed in detail [60].

The knockout of *DGCR8* leads to a global loss of mature miRNAs in mouse ESCs, mainly represented by non-canonical miRNAs [34]. In a knockout model of *DGCR8*, the evaluation of early stages of thyroid development detected a severe hypothyroidism with almost undetectable free T4, thyroid tissue disorganization, and few follicular structures [14]. Depletion of miRNAs by its turn results in severe proliferation deficiency and failure to silence the self-renewal program in mouse ESCs [58]. A study led by Kumar et al. [61], shows that the silencing of different components of the miRNA processing machinery decreases miRNA levels and results in a more pronounced phenotype. Interestingly, miRNA machinery knockdown leads to the activation of proto-oncogenes such as *K-RAS* and *c-MYC*, indicating that the expression of tumor-suppressor miRNAs is essential to protect cells from the activation of proto-oncogenes and to maintain cell differentiation [14]. It was previously demonstrated that *c-MYC* represses the transcriptional activity of tumor- suppressive miRNAs such as the *let*-7 family [28]. The global repression of miRNA maturation promotes cellular transformation and tumorigenesis [61]. miRNA processing-impaired cells form tumors with accelerated kinetics, translating into more invasive tumors [61].

The *DGCR8* mRNA dysregulation affects the miRNA machinery and, therefore, may have a role in TC [33,62]. Rodrigues et al. [33] reported overexpression of *DGCR8* mRNA in FTA, suggesting that *DGCR8* could play a role in maintaining the “normal” thyroid gland morphology. On the other hand, follicular-patterned TC (FTC and follicular subtype of PTC) displayed lower gene expression when compared to their normal counterpart. These results suggest that dysregulation in expression takes a role in thyroid tumorigenesis as dedifferentiation occurs, especially in follicular-patterned carcinomas [33].

### 1.5. DGCR8 E518K Hotspot Mutation

*DGCR8* localizes in human chromosome 22 at q11.2 region [10,33,62]. A recurrent mutation in *DGCR8* has been reported in TC: the c.1552 G>A, p.E518K, located in helix 1 of the first of two double-strand RNA-binding domains within DGCR8 [10,33,62]. The E518 residue is responsible by forming a critical hydrogen bond with the 2′ hydroxyl group of the pentose ring in the RNA molecule [10]. In silico modeling predicts that mutating amino acid 518 from glutamate to a lysine would likely reduce the affinity of RNA binding to DGCR8 [10]. The E518K variant reduces the expression of mature miRNA levels when compared to their normal counterpart, being accompanied by a corresponding accumulation of pri-miRNAs [10,34]. An aberrant profile of miRNA expression may have consequences for gene regulation at the post-transcriptional level [63]. Earlier studies reported that the geometry of the RNA-binding complex is not altered between DGCR8-wild-type (WT) and DGCR8-E518K, proving that the dysregulation in miRNA length is not due to shifted cutting sites due to an altered geometry of the RNA-protein complex [34]. The E518K mutation causes a reduction of critical miRNAs in tumors, which is consistent with the observation that the knockdown of *DGCR8* promotes tumor growth [64]. A recent study conducted by Condello et al. [63] reported that the mutational status of *DGCR8* rather than tumor histotype determines the global change in miRNA expression. The miR-30-c-2 is underexpressed in the presence of the E518K mutation, whereas some miRNAs such as miR-223 are not affected by the presence of this mutation [10]. In a previous study, all non-canonical miRNAs presented graded expression with the highest levels in *DGCR8*-KO cells, followed by *DGCR8*-E518K, and lowest levels in *DGCR8*-WT [34]. The moderate expression levels of miRNA in E518K cells indicate only a partial rescue of miRNAs [34]. Evaluations of miRNA target interactions revealed that *DGCR8*-E518K cells play a role in post-transcriptional control and signaling, as well as development processes and differentiation [34]. The fact that this mutation has a repercussion in the miRNA profile could be used as a therapeutic tool, as long as restoration of overexpressed or downregulated miRNAs occurs.

It was demonstrated by Rivera et al. [10] that *DGCR8*-E518K differentially expressed *KRAS* and *NRAS* mRNA in tumor tissue when compared to the normal counterpart. It has been described that for tumorigenesis to occur in this setting, the E518K should be accompanied by another somatic event: loss of heterozygosity (LOH) through copy number loss of the remaining WT *DGCR8* allele [10]. Although in other types of cancer where E518K mutation is found, other mutations affecting *DGCR8* are described. In the particular case of TC however, only the E518K mutation is reported [33,64]. As the loss of the WT allele is suggested for tumorigenesis, *DGCR8* seems to act as a tumor-suppressor gene for which the E518K allele confers transforming properties [34]. The ploidy of thyroid lesions has been extensively studied by several authors, being globally accepted that the presence of aneuploidy is an adverse prognostic factor in TC [65]. The deletion of chromosome 22 is of major interest in follicular tumorigenesis since it has been frequently found deleted in follicular carcinomas [65]. This two-hit theory seems to be also required in other miRNA biogenesis genes, like *DROSHA* and *DICER1*, in some specific types of tumor, where the hotspot mutation is always accompanied by another loss of function (LOF) mutation or LOH [66,67]. Previous studies reported that the biallelic alteration in *DGCR8*—the E518K missense mutation accompanied by LOH where the WT allele localizes—seems to be present not only in thyroid tumors, but also in Wilms’ tumors, schwannomatosis, and pineoblastomas [66,68]. A common miRNA profile is shared by E518K-mutated lesions suggesting that miRNA biogenesis is similarly impacted by this mutation in these distinct tumors [10].

The DICER1-syndrome was the first described cancer predisposition syndrome caused by impaired miRNA biogenesis. DICER1-syndrome usually harbors biallelic pathogenic variants: a germline LOF pathogenic variant in one allele that can occur in any domain, and a tumor-specific pathogenic somatic variant in exons encoding the RNase IIIb domain of the second allele [69]. DICER1-syndrome has been linked to pleuropulmonary blastomas, Wilms’ tumor, follicular nodular disease, thyroid cancer, and other neoplasias [9]. Following DICER1-syndrome, another syndrome caused by impaired miRNA biogenesis was recently described: a syndrome characterized by familial follicular nodular disease and schwannomatosis caused by a germline mutation in *DGCR8*, the E518K mutation [10]. Regarding Schwann cells, *DGCR8* is necessary for modulation of myelin formation and maintenance, just as it is for thyroid development and follicle formation [56].

The same mutation, *DGCR8* E518K, was reported in sporadic cases of follicular subtypes such as PTCs, FTCs, and PDTCs, where an additional LOF mutation or LOH seems to be required for carcinogenesis to happen [10,33,62]. In the reported sporadic PTC and FTC harboring the E518K mutation, *NRAS* mutation concomitantly was also detected; this is in agreement with *NRAS* mutations’ higher frequency in follicular-patterned tumors [33]. The resulting altered miRNA expression patterns are hypothesized to underlie tumor formation [66]. Beyond *DGCR8*, somatic *DICER1* mutations are reported in follicular-patterned lesions of the thyroid which underlines the importance of miRNA processing genes in follicular-patterned lesions [62,70]. In Wilms’ tumors and pineoblastoma, somatic mutations in miRNA biogenesis genes (*DROSHA*, *DGCR8*, *DICER1)* or in genes involved in the degradation of miRNA (*DIS3L2*) have also been reported [71]. This opens new questions about other miRNA biogenesis genes, besides *DICER1* and *DGCR8*, which may have a role in thyroid tumorigenesis.

So far, the findings comprising *DGCR8* strengthen the association between abnormal miRNA processing and the development of TC, with a highlighted role in follicular-patterned TC. It is suggested that the *DGCR8* mutation influences the tumor progression or invasive behavior without driving the tumor formation per se [33,62].

## 2. Conclusions

The miRNAs are key regulators of gene expression in many cellular processes. They are of particular interest in thyroid gland formation, where a crucial role of the miRNA biogenesis pathway is a fate determinant for accurate development of thyroid follicles and proper hormone synthesis. Along with this alteration in the thyrocyte physiology, we have altered expression profiles of the cell’s miRNAs and, consequently, novel transcriptomic programs emerging. These programs or expression profiles serve many ends and can be used to differentiate between normal tissues, benign, and malignant tumors, and eventually to predict patient outcomes. The link between the malfunction of the miRNA machinery and thyroid disease was first observed in DICER1-syndrome. This was the first cancer predisposition syndrome caused by a disruption in miRNA biogenesis. It was only a matter of time before other important components would start to be investigated. A recurrent mutation in a component of miRNA machinery, the *DGCR8*, has identified the E518K hotspot mutation; it has been associated with TCs by several authors. We are now progressing in the characterization of the *DGCR8* impact in thyroid neoplasia and not only mutations but also dysregulation of its expression is detected; the latter being especially important in follicular-patterned TC and corroborating the importance of miRNA biogenesis, as well as its components in the follicular differentiation of the thyroid. The DGCR8 comprehension in thyroid disease is only in its infancy and it is expectable that the impact of *DGCR8* in TC and its consequent miRNA expression dysregulation could direct further studies in order to elucidate what the contribution of this alteration is in the thyroid gland, as well described in Wilms’ tumors, and if it opens novel potential therapeutics opportunities for TC, e.g., using RNA-based miRNA inhibitors or supplements to modulate the cancer cells.
